# 2020 List of Reviewers

**DOI:** 10.1117/1.NPh.8.1.010102

**Published:** 2021-01-22

## Abstract

Thanks to reviewers who served Neurophotonics in 2020.

*Neurophotonics* would like to sincerely thank the following individuals who served as reviewers in 2020. The success of our publication hinges on the voluntary contributions of time and energy put forth by these professionals.

Ardalan Aarabi
Yoav Adam
George Alexandrakis
Anna Letizia Allegra-Mascaro
Juanita Anders
Markus Axer
Wesley Baker
Gemma Bale
Adam Bauer
Giulio Bicciato
Pablo Blinder
Edward Boyden
Sabrina Brigadoi
David Busch
Marco Canepari
Stefan Carp
Jonathan Cayce
Mykyta Chernov
Antonio Chiarelli
Bernard Choi
Myunghwan Choi
Riccardo Cicchi
Emma Condy
Christian Crouzet
Helga De Oliveira Miguel
Massimo De Vittorio
Hamid Dehghani
Damon DePaoli
Andrew Dunn
Adam Eggebrecht
Sinem Erdogan
Qianqian Fang
Parisa Farzam
Pietro Ferraro
Rodrigo Forti
Tsukasa Funane
Jessica Gemignani
Antoine Godin
Gorm Greisen
Edgar Guevara
Yun He
Joy Hirsch
Keum-Shik Hong
Jie Hui
Maria Iennaco
Takahiro Ikeda
Chang-Hwan Im
Jingjing Jiang
Bjoern Kemper
Kivilcim Kilic
Beop-Min Kim
Lisa Kobayashi
Jonghwan Lee
Lei Li
Chao Liu
Chengbo Liu
Sylvie Lorthois
Chris McKnight
Shalin Mehta
Miriam Menzel
Daniel Milej
Yukifumi Monden
Noman Naseer
Eric Newman
Thien Nguyen
Jack Noah
Sergio Novi Junior
Yihong Ong
Felipe Orihuela-Espina
Adam Packer
Ashwin Parthasarathy
Stephane Perrey
Daqing Piao
Paola Pinti
Franck Plouraboue
Luca Pollonini
Dmitry Postnov
Sotiris Psilodimitrakopoulos
Kyle Quinn
Jessica Ramella-Roman
Allan Reiss
Mitchell Robinson
Shuzo Sakata
Michelle Sander
Hendrik Santosa
Hiroki Sato
Joao Sato
Giovanni Scavariello
Christopher Schaffer
Felix Scholkmann
Anna Schueth
Dietrich Schweitzer
Janaka Senarathna
Myeongsu Seong
Sotaro Shimada
Amir Shmuel
Shy Shoham
Yousheng Shu
Spencer Smith
Marcelo Sousa
Vivek Srinivasan
Keith St. Lawrence
Jillian Stobart
Stephanie Sutoko
Toshimitsu Takahashi
Kevin Takasaki
Johnny Tam
Jianbo Tang
Qinggong Tang
Tong Boon Tang
Tomomi Tani
Glen Tellis
Alexander Thompson
Martin Thunemann
Lei Tian
Alessandro Torricelli
Francois Treussart
Daniel Tso
Daisuke Tsuzuki
Chris Valdez
Jenu Varghese-Chacko
Alexander von Luehmann
Heidrun Wabnitz
Alex Walsh
Hui Wang
Dongli Xu
Jiarui Yang
Weijian Yang
Mohammad Yaseen
Konstantin Yashin
Ji Yi
Zhen Yuan
Xian Zhang
Hubin Zhao
Chao Zhou

